# Creating an Animal Model of Tendinopathy by Inducing Chondrogenic Differentiation with Kartogenin

**DOI:** 10.1371/journal.pone.0148557

**Published:** 2016-02-05

**Authors:** Ting Yuan, Jianying Zhang, Guangyi Zhao, Yiqin Zhou, Chang-Qing Zhang, James H-C. Wang

**Affiliations:** 1 MechanoBiology Laboratory, Department of Orthopaedic Surgery, University of Pittsburgh School of Medicine, Pittsburgh, PA, United States of America; 2 Department of Orthopaedic Surgery, Shanghai Sixth People's Hospital Affiliated to Shanghai Jiaotong University, Shanghai, China; Louisiana State University, UNITED STATES

## Abstract

Previous animal studies have shown that long term rat treadmill running induces over-use tendinopathy, which manifests as proteoglycan accumulation and chondrocytes-like cells within the affected tendons. Creating this animal model of tendinopathy by long term treadmill running is however time-consuming, costly and may vary among animals. In this study, we used a new approach to develop an animal model of tendinopathy using kartogenin (KGN), a bio-compound that can stimulate endogenous stem/progenitor cells to differentiate into chondrocytes. KGN-beads were fabricated and implanted into rat Achilles tendons. Five weeks after implantation, chondrocytes and proteoglycan accumulation were found at the KGN implanted site. Vascularity as well as disorganization in collagen fibers were also present in the same site along with increased expression of the chondrocyte specific marker, collagen type II (Col. II). *In vitro* studies confirmed that KGN was released continuously from KGN-alginate *in vivo* beads and induced chondrogenic differentiation of tendon stem/progenitor cells (TSCs) suggesting that chondrogenesis after KGN-bead implantation into the rat tendons is likely due to the aberrant differentiation of TSCs into chondrocytes. Taken together, our results showed that KGN-alginate beads can be used to create a rat model of tendinopathy, which, at least in part, reproduces the features of over-use tendinopathy model created by long term treadmill running. This model is mechanistic (stem cell differentiation), highly reproducible and precise in creating *localized* tendinopathic lesions. It is expected that this model will be useful to evaluate the effects of various topical treatments such as NSAIDs and platelet-rich plasma (PRP) for the treatment of tendinopathy.

## Introduction

Tendinopathy is a prevalent tendon disorder that affects a large proportion of people in both athletic and occupational settings involving repetitive motion. Patients with tendinopathy often experience a number of debilitating symptoms including pain, stiffness or tenderness, edema and swelling in the injured tendon area [1]. However, the current treatment options for tendinopathy are largely palliative because the mechanisms causing the tendon disorder are not well understood [2].

Typically, tendinopathy is studied using samples from patients who choose surgical interventions to alleviate tendinopathy symptoms [3–6]. While much of our understating of tendinopathy stems from the histological analysis of the tendon samples removed during surgery, variations in the etiology of the disease in each patient has made it difficult to devise an effective treatment modality for the tendon disease. Thus, animal models of tendinopathy are required to investigate the cellular and molecular mechanisms regulating the tendon disorder to devise effective treatment protocols. Moreover, to develop better treatment options for tendinopathy, it is essential to have a reproducible, cost-effective animal model of tendinopathy that will allow evaluation of the tendon disease progression and enable development of better treatment options.

However, it is challenging to develop a single model that exhibits all features of tendinopathy, which is now considered to be a spectrum of tendon disorders resulting from multiple etiologic factors [7]. Common methods used to create animal tendinopathy models are treadmill running and biochemical injections [8,9].

Treadmill running was the preferred method to create tendinopathy models because mechanical loading is considered to be a major etiologic factor of tendinopathy in specific load-bearing tendons, such as patellar and Achilles tendons. Besides, this method is also "physiological" in the sense that it creates tendinopathy that occurs naturally. Different treadmill running protocols (uphill, downhill, etc.) have been used to induce tendinopathic features like disorganization and disintegration of collagen fibers [10] [11], microtearing and increase in glycosaminoglycan content [12], and altered tendon cell morphology from elongated to round shape in the Achilles tendons [13]. Repetitive mechanical loading also caused tendon sub-failure injury in rat patellar tendons [8]. Moreover, our recent study on over-use tendinopathy suggested that degenerative changes in tendons may be caused by the aberrant differentiation of tendon stem/progenitor cells (TSCs) in response to excessive mechanical loading placed on the tendons [9].

While the mechanical loading-induced tendinopathy in animal models are physiologically relevant, they have serious limitations because it is time-consuming and costly to create these models. Additionally, the tendinopathic lesions induced by mechanical loading may vary among different animals and in different tendon regions of the same animals. Therefore, it is highly desirable to create an animal model in a more convenient, efficient and reproducible fashion.

Injection is another commonly used method to create animal models of tendinopathy. In the injection-induced tendinopathy model, tendon degeneration is achieved by injecting chemicals or bio-factors into the tendons. These include cell-activating factors (CAF) [14], prostaglandins [15,16], collagenase [17,18] and TGF-β1[19]. Although these injection models can capture certain features of tendinopathy such as inflammation, disorganization of collagen matrix and hyper-vascularity, diffusion of the injected "bio-agents" into the surrounding area often poses a challenge to create "clean" and *localized* tendinopathic lesions inside tendons. For example, PGE_1_ and PGE_2_ [15] leakage into areas around the treated region creates unintentional inflammation in these regions.

Therefore in this study, our aim was to create a localized animal model of tendinopathy by implanting fine beads of a bio-compound called kartogenin (KGN) into rat tendons. KGN is a small heterocyclic compound that can stimulate endogenous stem/progenitor cells such as mesenchymal stem cells to differentiate into chondrocytes in mice [20]. In tendinopathy patients, chondrocyte-like cells have been demonstrated in the degenerated regions where increased glycosaminoglycan was present [5,6,21] and upregulated expression of proteoglycans, aggrecan and biglycan transcripts was detected [4]. Therefore, in this study we attempted to create a tendinopathy model by implanting fine beads of alginate containing KGN into rat tendons.

## Methods

### Ethics statement

The University of Pittsburgh IACUC approved all experimental protocols for the use of rats in this study.

### Preparation of KGN beads

**Fig 1:**
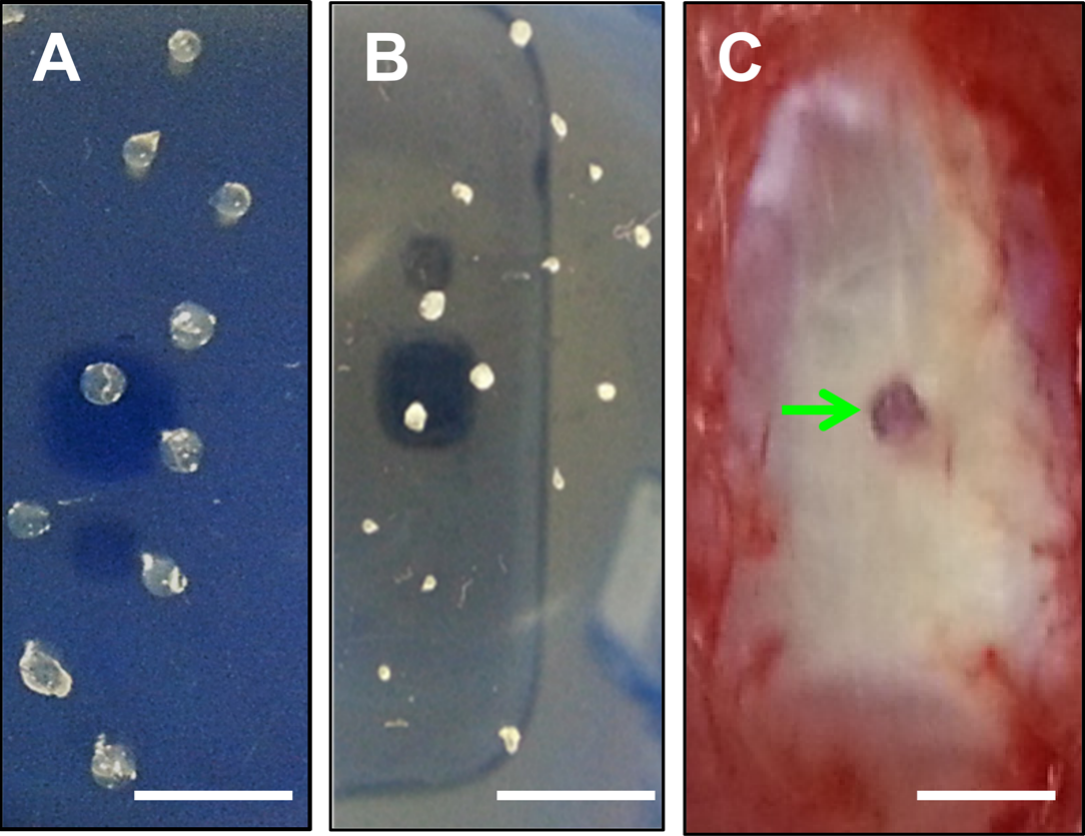
KGN bead implantation in rat Achilles tendons *in vivo*. A. KGN beads were prepared by squeezing drops of KGN+DMSO+alginate solution through a pipet into a calcium chloride solution. B. KGN beads air dried for 48 hrs. Diameter of the dried beads was 169 ± 32 μm. C. Rat Achilles tendon with the implanted bead. KGN implanted rats were allowed free cage activities until further analyses. Arrow (C) points to the KGN bead implanted site. Bar– 1 mm.

KGN beads were prepared by dissolving 5 mg KGN in 0.3 ml DMSO to make a 50 mM KGN stock solution, which was diluted in double distilled water to obtain a 5 mM KGN working solution. From this, 400 μl was added to 1 ml of 2% alginate to form a 1.5 mM KGN-DMSO-alginate suspension. The KGN-DMSO-alginate mixture was then drawn into a pipette and squeezed to obtain drops that were allowed to fall into a 2% calcium chloride solution to stimulate gel formation. Each drop contained about 5 μL of KGN-DMSO-alginate and therefore 2.5 μg KGN in each bead (**Fig 1A**). Similarly, control beads containing only DMSO-alginate were also prepared such that each bead contained 2.85% DMSO, which is identical to the amount of DMSO present in each KGN bead. All beads were air dried for 48 hrs before use (**Fig 1B**).

### *In vivo* animal experiments

To determine the chondrogenic potential of KGN *in vivo*, we implanted KGN beads into 8–12 weeks old female Sprague Dawley (SD) rats. Twelve rats were used for this experiment and were assigned randomly to one of two groups: KGN bead implanted group or DMSO bead implanted group. After anesthetizing rats by inhaling 1–3% isoflurane, one KGN bead or DMSO bead was implanted into a small puncture (< 0.5 mm in diameter) created in the lateral strand of the Achilles tendons 5 mm proximal to the calcaneal insertion and sutured (**Fig 1C**). Rats were allowed unrestricted activities in cages and sacrificed 5 weeks after implantation. Their Achilles tendons were collected for gross inspection and further analyses. Note that the amount of DMSO used in this study was too low to induce cytotoxic effects on cells. It has been shown that low DMSO concentrations (< 10%) do not cause cytotoxic changes in enterocyte-like cells *in vitro* [22].

### Histochemical analyses

For histochemical analysis, Achilles tendons were harvested from the KGN and DMSO bead implanted rats after 5 weeks and were immersed in frozen section medium (Neg 50; Richard-Allan Scientific; Kalamazoo, MI) in pre-labeled base molds. The tendons were then flash-frozen by placing in 2-methylbutane chilled in liquid nitrogen. The frozen tissue blocks were stored at -80°C until required or used immediately. Each frozen tissue was cut into 8 μm thick sections and placed on glass slides to dry overnight at room temperature. The sections were then fixed in 4% paraformaldehyde for 15 min and stained with hematoxylin/eosin (H&E). Additional tendon sections were also stained with Safranin O/fast green and Alcian blue/fast nuclear red according to our protocols [9].

### Immunofluorescence staining

To further characterize the tendinopathic lesions induced by KGN, we examined neovascularization and chondrogenesis in the KGN or DMSO bead implanted tendons by immunostaining with anti-CD31 and anti-collagen type II antibodies. Briefly, the frozen Achilles tendons from the KGN or DMSO beads implanted groups were cut into 8 μm thick sections and fixed in acetone for 5 min at room temperature. After washing in PBS and treating with blocking one solution (Nacalai Tesque Inc. Kyoto, Japan), the tissue sections were incubated with anti-CD31 (1:300) primary antibody (Abcam, Cambridge, MA) overnight at 4°C. Then, slides were rinsed 3 times in PBS and incubated with Cy3-conjugated secondary antibody. To determine chondrogenesis, the tissue sections were fixed with 4% paraformaldehyde in PBS for 15 min and incubated with mouse anti-collagen type II (1:300) primary antibody (Cat. #CP18-100UG; Millipore, Billerica, MA) at room temperature for 2 hrs. After washing three times in PBS, sections were incubated in FITC-conjugated goat anti-mouse IgG (1:500) at room temperature for 2 hrs. Excess secondary antibodies were removed by washing in PBS, followed by staining cellular nuclei with Hoechst fluorochrome 33342 (H33342). Finally, stained sections were observed through a fluorescence microscope (Nikon eclipse microscope, TE2000-U, Nikon Instruments Inc., Melville, NY, USA).

### KGN release experiment *in vitro*

Twenty KGN-alginate beads obtained as above were weighed and incubated in 1 ml PBS at 37°C with continuous rotation using a micro-hybridization incubator (Robbines Scientific, Sunnyvale, CA). Every 10 min for 70 min, the beads were separated from the solution by a vacuum filtration funnel, dried gently using a soft tissue paper and weighed to record bead weight. The amount of KGN released from the beads into the PBS solution was also determined every 10 min for 70 min by obtaining an aliquot of the PBS using high performance liquid chromatography (HPLC; Agilent Technologies, Santa Clara, CA) and a standard curve (R^2^ = 0.996). This experiment was performed three times with 3 replicates in each treatment.

### TSC isolation and Safranin O staining

To determine whether KGN-induced chondrogenesis in rat tendons is mediated through tendon stem/progenitor cells (TSCs), we performed the following *in vitro* study. Briefly, 5 Sprague Dawley (SD) rats (8–10 weeks old, female) were used in the experiments to obtain Achilles tendons (n = 10). Then, after discarding the tendon sheath and surrounding paratenon, the tendons were minced into small pieces. A 0.5 ml solution containing collagenase type I (3 mg/ml) and dispase (4 mg/ml) was then used to digest 10 mg of the minced tendon tissue at 37°C for 1 hr. Cells from this digest were recovered by centrifugation at 1,500 g for 15 min and the cell pellet was re-suspended and cultured in Dulbecco’s modified Eagle medium (DMEM, Lonza, Walkersville, MD) containing 20% fetal bovine serum (FBS), 100 U/ml penicillin and 100 μg /ml streptomycin (Atlanta Biologicals, Lawrenceville, GA) in T25 flasks. TSC colonies were observed after about 2 weeks in culture. These colonies were sub-cultured and used for the *in vitro* assays. Prior to experimentation, TSCs were characterized for the presence of stem cell markers, nucleostemin, Oct-4 and SSEA-1, as previously described [9].

TSCs at passage 1–2 were seeded in 6-well plates at a density of 2× 10^5^ per well and cultured for 21 days in DMEM (Dulbecco’s modified Eagle’s medium) plus 10% fetal bovine serum (FBS) with various concentrations of KGN (0 nM, 1 nM, 10 nM, 100 nM, 1 μM and 10 μM). The medium was changed every 3 days and after 21 days, cells were stained with Safranin O solution (Sigma, St. Louis, MO) as previously described [23]. The experiment was performed three times with 3 replicates in each treatment.

### qRT-PCR analysis of TSCs *in vitro*

Expression of chondrocyte related genes, aggrecan, collagen type II (Col. II) and Sox-9, in TSCs treated with various KGN concentrations was determined using standard qRT-PCR protocols [23]. Rat-specific primers for the chondrocyte markers (**Table 1**) were adopted from previous studies and synthesized by Invitrogen (Invitrogen, Grand Island, NY). The housekeeping gene, glyceraldehyde 3-phosphate dehydrogenase (GAPDH), served as an internal control. Relative gene expression levels were estimated using the 2^-ΔΔCt^ method [24]. The experiment was performed three times with 3 replicates in each treatment.

**Table 1:** Rat RT-PCR primer sequences.

Gene	Forward	Reverse
GAPDH	5’-AACTCCCTCAAGATTGTCAGCA-3’	5’-TCCACCACCCTGTTGGCTGTA-3’
Aggrecan	5’-TCAGGAACTGAACTCAGTGG-3’	5’-GCCACTGACTTCCACAGA-3’
Collagen-II	5’-AGTGGAAGAGCGGAGACTA-3’	5’-GACAGGCCCTATGTCCACAC-3’
SOX-9	5’-AGCGACAACTTTACCAG-3’	5’-GGAAAACAGAGAACGAAAC-3'

### Statistical analyses

One-way ANOVA was used, followed by Fisher's Least Significant Difference (LSD) test for multiple comparisons. Differences between two groups were considered significant when *P*-values were less than 0.05.

## Results

### Effects of KGN bead implantation on rat Achilles tendons *in vivo*

**Fig 2:**
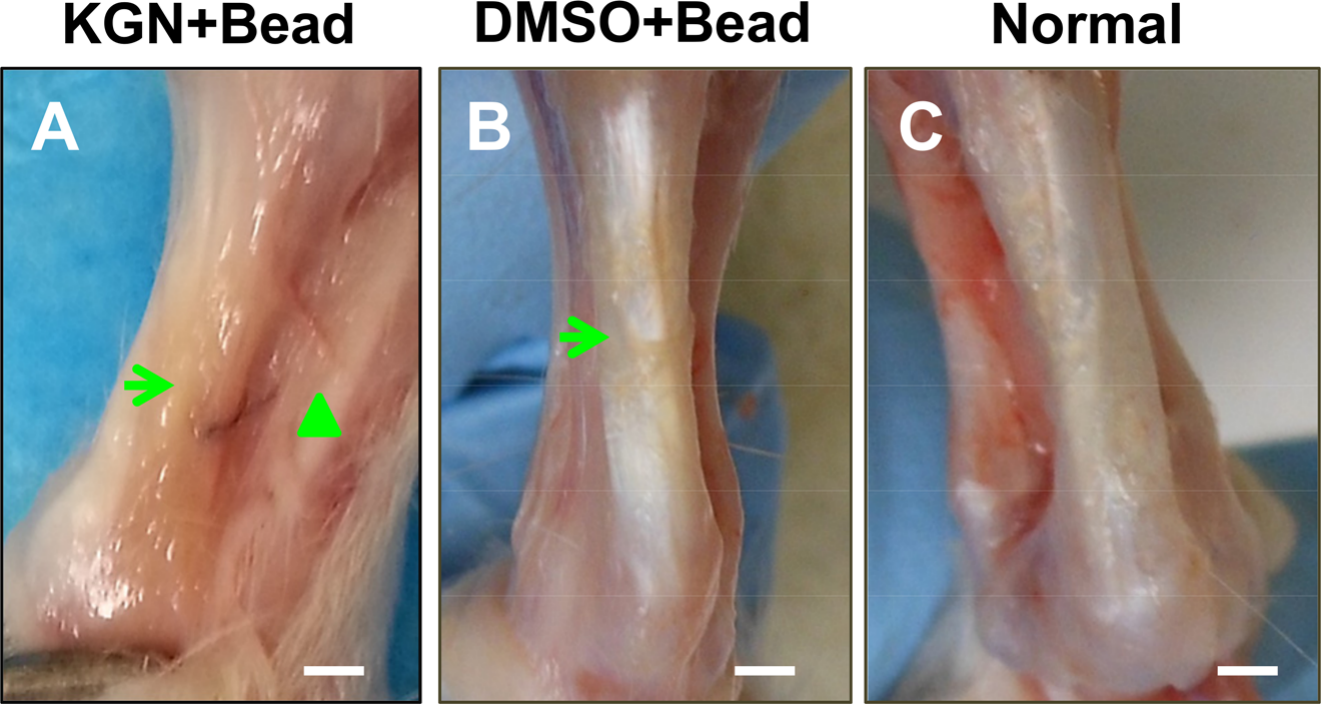
Gross appearance of rat Achilles tendons after KGN bead implantation *in vivo*. Five weeks after implantation, KGN bead implanted Achilles tendons showed thickened peritenon (A, triangle points to the skin), while the DMSO bead implanted control Achilles tendons had an apparent normal structure (B) similar to the normal rat Achilles tendon (C). Arrows indicate the site of KGN bead or DMSO bead implanted area. Bar– 1 mm.

**Fig 3:**
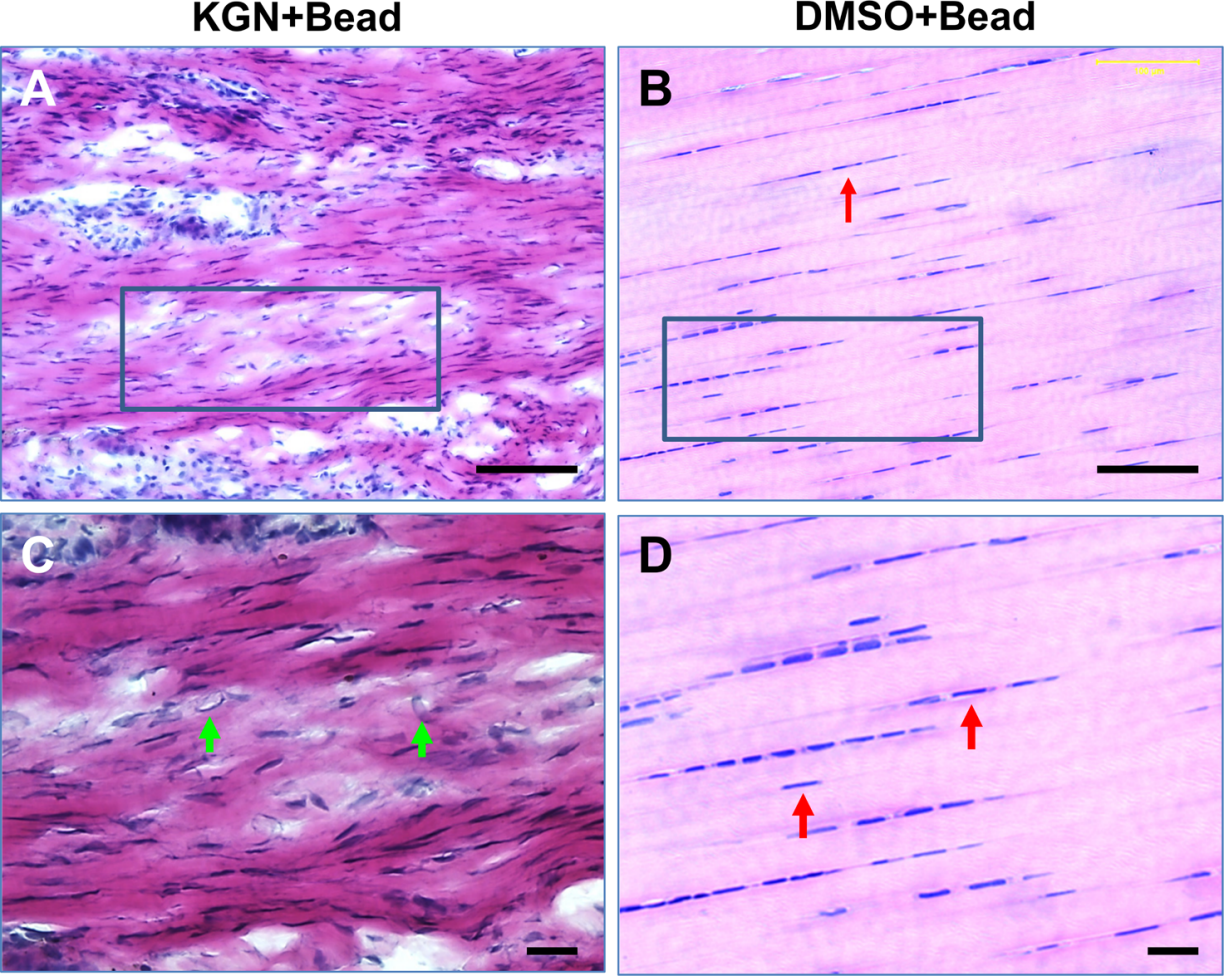
H&E staining of the KGN-bead implanted tendons. Five weeks post-implantation of KGN beads (A), round chondrocyte-like cells (boxed) were observed in the KGN bead implanted tendons only. The boxed area in A is enlarged in C. Green arrows indicate chondrocyte-like cells. In contrast, DMSO-bead implantation did not affect tendon structure (B). The rectangular area in B is enlarged in D. Red arrows indicate normal tendon cells. Bar– 100 μm.

Towards creating an animal model of tendinopathy, we first determined the effects of KGN bead implantation on rat Achilles tendons. Five weeks after KGN bead implantation, thickening of the paratenon tissues occurred (**Fig 2A**); in contrast, the DMSO bead implanted control tendons had a white glistening appearance (**Fig 2B**) similar to the gross appearance of intact tendons (**Fig 2C**). Examination of the H&E stained tendon sections revealed typical tendinopathy features such as hypercellularity and collagen disorganization only in the KGN bead implanted tendon sections (**Fig 3A and 3C**). However, the DMSO bead implanted control tissues appeared normal with no signs of disruption in the collagen structure or increased cellular contents (**Fig 3B and 3D**). In addition, the KGN bead implanted tendons had round cells (**Fig 3A and 3C**) unlike the DMSO bead implanted tendons that had a more elongated cell structure typical of normal tendon cells (**Fig 3B and 3D**).

**Fig 4:**
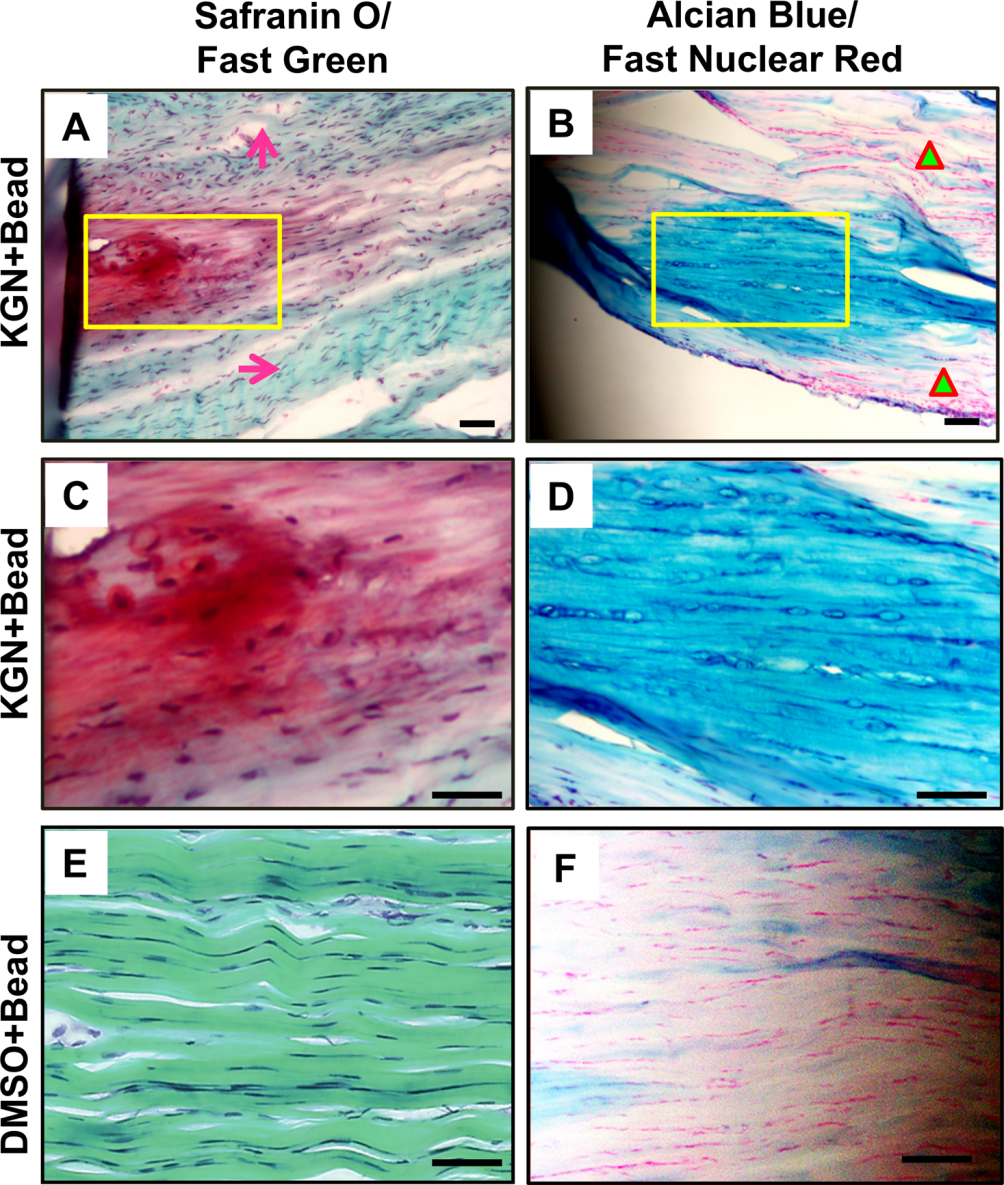
Safranin O/Fast green (A, C, E) and Alcian blue/Fast nuclear red (B, D, F) staining of the KGN bead implanted sites in rat Achilles tendons *in vivo*. Note that C is an enlargement of the rectangled region in A. Arrows point to the area outside the KGN-bead implantation site that appears normal. D is an enlargement of the yellow box in B. Triangles indicate the normal areas outside the KGN-bead implantation site. Positive staining was observed only in the KGN bead implanted sites (A, B, C, D) indicating the presence of glycosaminoglycan rich matrix representing chondrogenesis only at these sites. The DMSO bead implanted tissue sections stained minimally for these stains (E, F). Bar– 100 μm.

**Fig 5:**
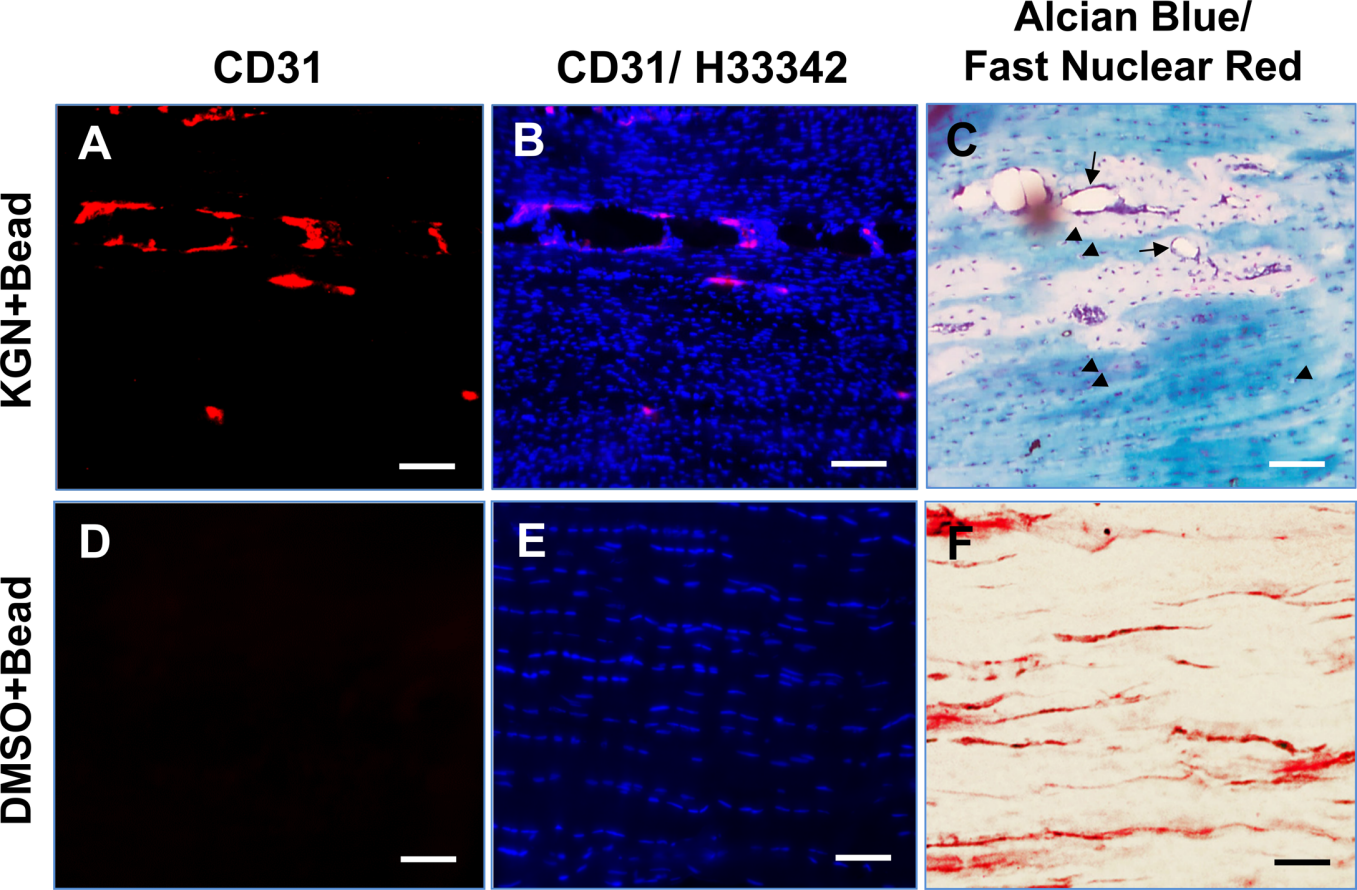
Immunohistochemical staining of CD31 to visualize neovascularization in KGN bead implanted rat Achilles tendons. CD31 positive staining (red) was observed (A, B) along the Alcian blue and fast red stained regions (C) in the KGN bead implanted Achilles tendon. Rest of the tendon was negative for CD31. Arrowheads indicate the chondrocyte-like cells and arrows point to the newly formed blood vessels. These vessels were positive for CD31. The DMSO bead implanted tendons were negative for CD31 staining (D, E), chondrocyte-like round cells and neovascularization (F). A, B–Red indicates CD31 staining; B, E–Blue indicates nuclear staining by Hoechst 33342. C, F–Alcian blue and fast red staining. Bar– 100 μm.

**Fig 6:**
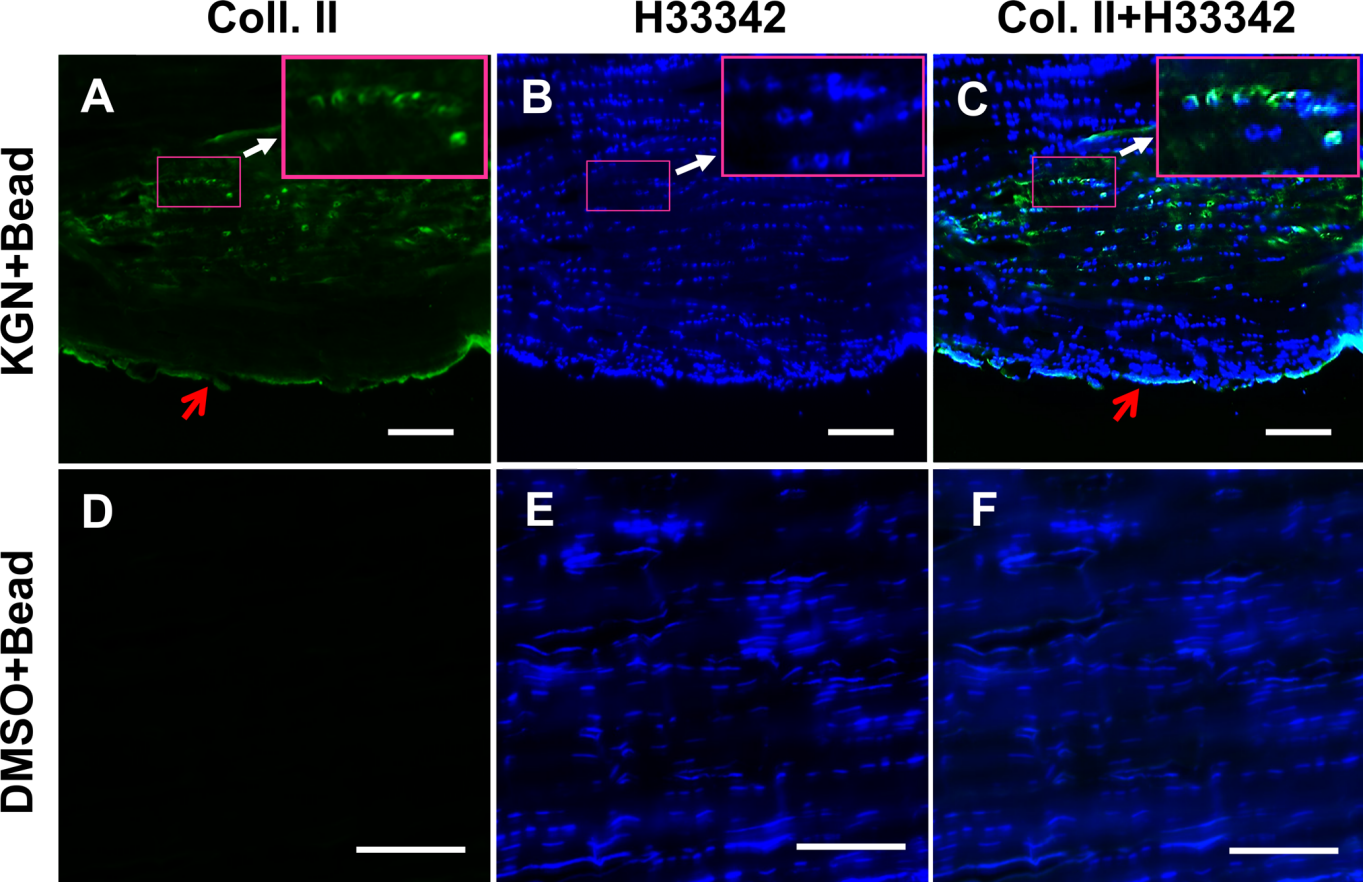
Immunohistochemical staining of collagen type II (Col. II) in KGN bead implanted rat Achilles tendons. The KGN bead implanted site in the Achilles tendon stained positive for Col. II (green in A, C–pink box; large boxes are enlargements of corresponding small boxes). Red arrows point to staining artifacts (green along the tendon edge), whereas the surrounding tissues and the DMSO bead implanted tissues (D, F) stained negative for Col. II. A, D–Green indicates positive Col. II staining; B, E–Blue indicates nuclei stained with Hoechst fluorochrome 33342; C, F–Overlay of Col. II and Hoechst staining. Bar– 150 μm.

Furthermore, staining for Safranin O/fast green and Alcian blue/fast nuclear red was positive only in the rat Achilles tendons that received the KGN bead implantation (**Fig 4A, 4B, 4C and 4D**) but not in the DMSO bead implanted controls (**Fig 4E and 4F**) indicating the presence of extensive amounts of proteoglycans only in the KGN-bead implanted tendons. More importantly, staining was observed only in the KGN bead implanted region but not in the surrounding fibril tissues and other sites in the neighboring areas (arrows and triangles in **A, B**). In addition, immunostaining of the KGN bead implanted areas with anti-CD31 antibody revealed positive staining only in the KGN bead implanted areas (**Fig 5A and 5B**) that overlapped with Alcian blue and fast red staining in the region (**Fig 5C**). CD31 and Alcian blue positive regions were not observed in the DMSO bead implanted control tendons (**Fig 5D, 5E and 5F**). Immunohistochemical staining for the chondrocyte marker, Col. II, was also intense in the KGN bead implanted rat Achilles tendons (**Fig 6A and 6C**) but not in the DMSO control group (**Fig 6D and 6F**). These chondrocyte positive regions also showed presence of numerous cellular nuclei (**Fig 6B**) when compared to DMSO control **(Fig 6E**). Together, these findings indicate that KGN induces localized chondrogenesis with neo-vascularization in rat Achilles tendons.

### KGN release from KGN-alginate beads *in vitro*

**Fig 7:**
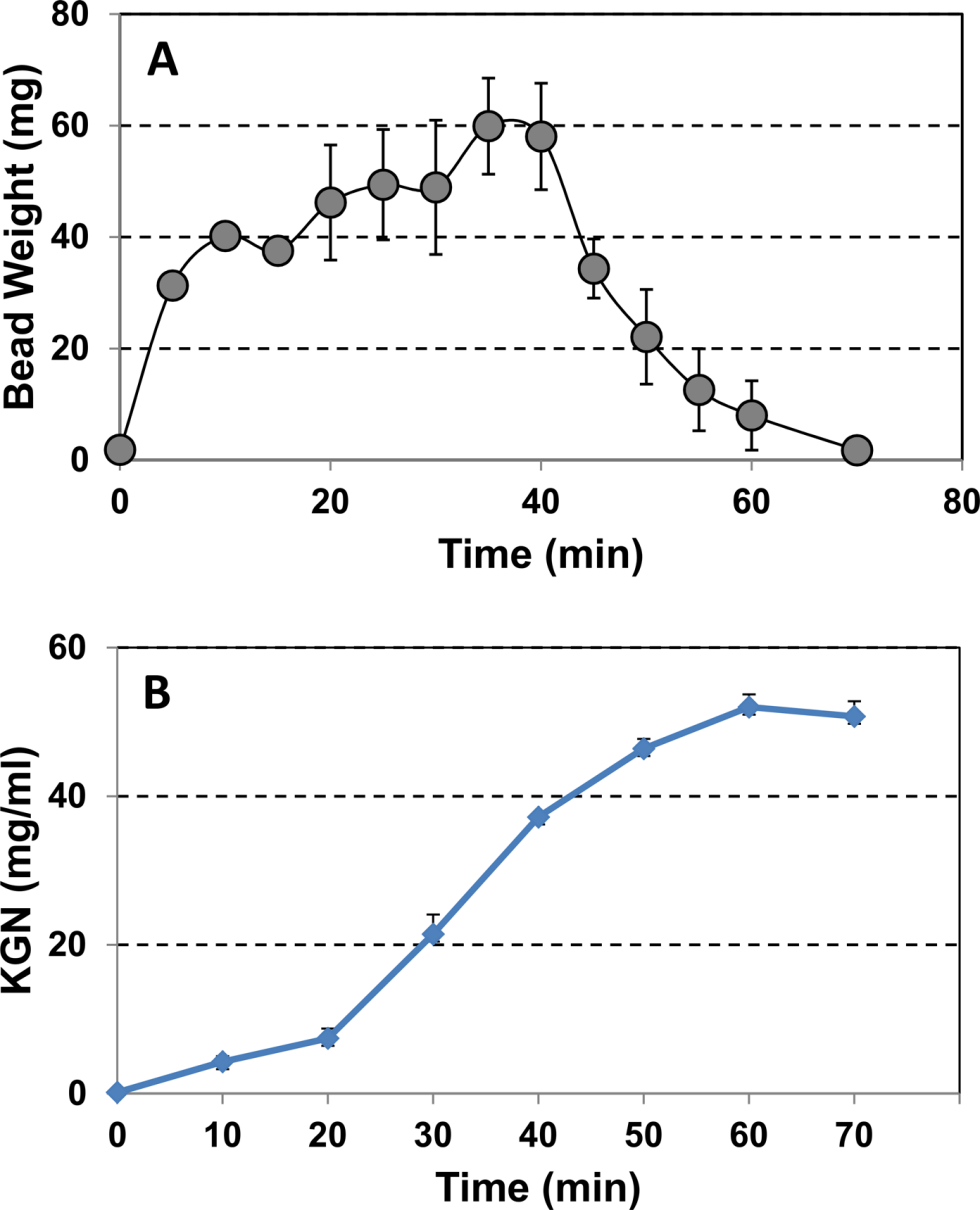
Release of KGN from KGN-alginate beads during their degradation *in vitro* at 37°C in PBS at various time points. (A) Changes in the weight of KGN-DMSO-alginate beads during degradation. (B) KGN release kinetics during degradation of the KGN-DMSO-alginate beads.

To assess how KGN was released into the surroundings after implantation of KGN-beads into the tendon, we performed an *in vitro* experiment. We found that the weight and size of alginate beads in PBS solution at 37°C increased and reached the maximum level at 40 min (**Fig 7A**) likely due to absorption of PBS solution by the dry beads. However, both weight and size of the beads started declining from 40 min and the beads completely disappeared by 70 min (**Fig 7A**). Furthermore, the concentration of KGN in the PBS solution increased in a time-dependent manner with a marginal increase in the first 20 min followed by a remarkable increase from 30–60 min after which there was a slight decline at 70 min (**Fig 7B**). Almost 100% of the KGN was released from the KGN-alginate beads at 70 min thus aligning well with the complete degradation of the KGN beads at this time point (**Fig 7A and 7B**).

### Effect of KGN on chondrogenic differentiation of TSCs *in vitro*

**Fig 8:**
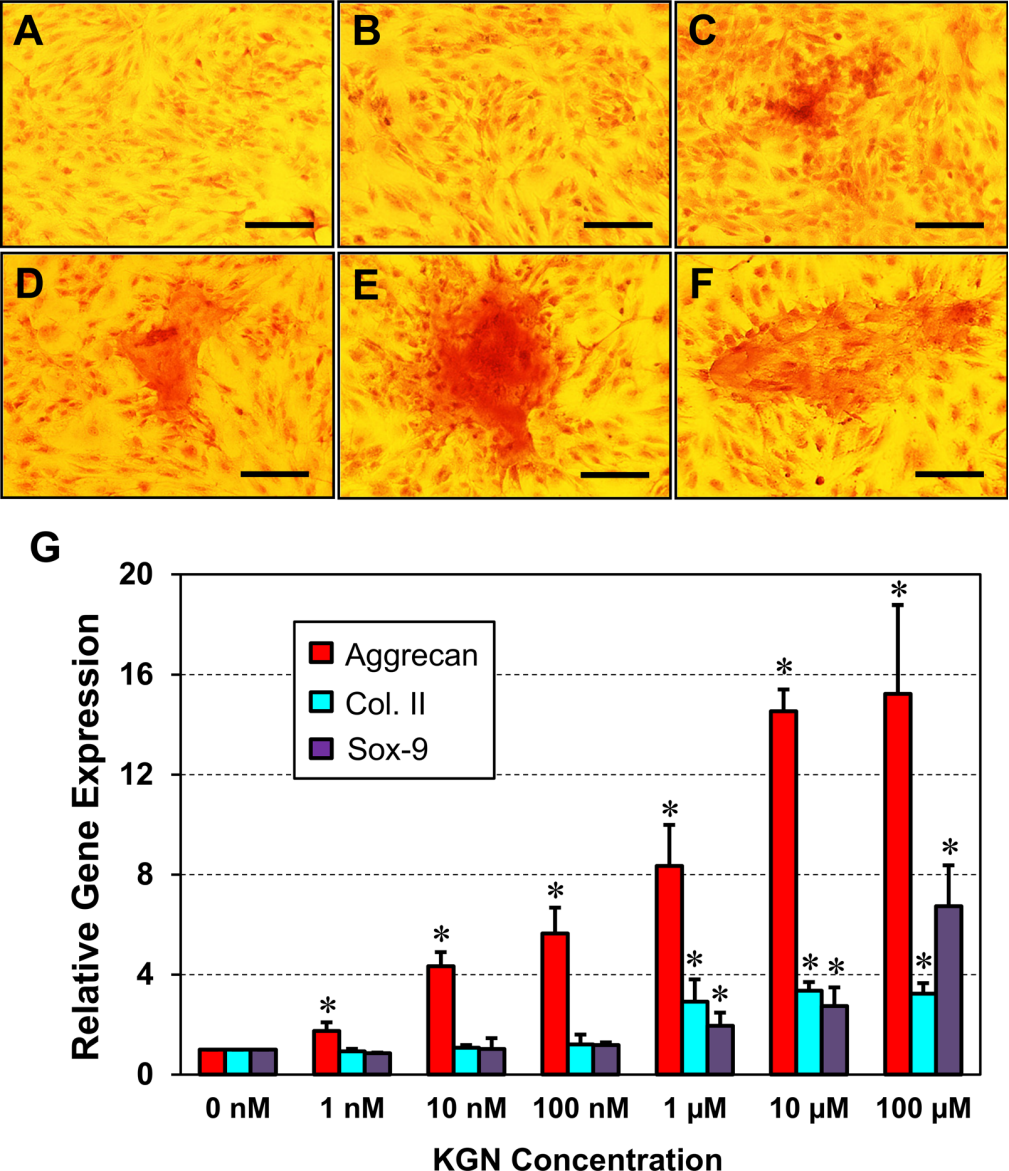
Safranin O staining of KGN treated rat Achilles TSCs *in vitro* (A-F). TSCs treated with various KGN concentrations for 21 days were stained with Safranin O. Red color indicates the increase in the accumulation of glycosaminoglycan-rich matrix in cell cultures in a KGN concentration-dependent manner (KGN concentration: A– 0 nM; B– 1 nM; C– 10 nM; D– 100 nM; E– 1 μM; F– 10 μM). Gene expression analysis in KGN treated TSCs *in vitro* (G). Rat TSCs were cultured in growth medium containing various KGN concentrations for 72 hrs, followed by RNA isolation and qRT-PCR to determine chondrocyte specific gene expression. KGN up-regulated the expression of aggrecan, Col. II and Sox-9. Values represent mean ± SD. Asterisks indicate significant differences (P < 0.05) between the KGN treated and control groups (0 nM). Bar– 100 μm.

To understand the cellular mechanism by which KGN induces the formation of cartilage-like tissues *in vivo*, we performed *in vitro* experiments where TSCs from rat Achilles tendons were treated with various concentrations of KGN. The results showed that glycosaminoglycan matrix staining in KGN-treated TSCs increased in a KGN dose-dependent manner with the highest accumulation in 10 μM KGN treated TSCs (**Fig 8A–8F**).

These results were further corroborated by qRT-PCR analysis. KGN up-regulated three chondrocyte specific genes, aggrecan, Col. II and Sox-9 in TSCs treated with KGN *in vitro*. When compared to the controls treated with DMSO (0 nM), KGN treatment significantly up-regulated all three chondrocyte genes in a dose-dependent manner (**Fig 8G**). Even at the lowest dose (1 nM) aggrecan levels increased 0.7-fold and steadily rose thereafter with increasing KGN concentration. At the highest 100 μM concentration the expression of aggrecan reached the maximum level, which was 14-fold higher than the control group without KGN treatment. However, Col. II and Sox-9 expression increased significantly only at 1 μM KGN concentration and reached up to 2.2-fold (Col. II) and 5.7-fold (Sox-9), respectively at the highest KGN dose (100 μM) (**Fig 8G**).

## Discussion

Animal models of tendinopathy are essential not only to study its developmental mechanisms but also to devise new treatment options for tendinopathy. However, the existing models using long term mechanical loading on the animal tendons, typically in the form of treadmill running are lengthy and expensive to produce, and may vary highly among animals. On the other hand, the models created by injection of various "bio-agents" create diffused tendinopathic lesions throughout the tendon and are not representative of real tendinopathic lesions, which are typically localized. Additionally, "bio-agents" like PGE_1_, PGE_2_, CAF and TGF-β1 [15,19,25] tend to be unstable thus creating potential variable results. In this study, we addressed these issues and developed a novel localized animal model of tendinopathy by implanting fine KGN-beads into rat Achilles tendons. Our rationale to use KGN-bead implantation was to contain KGN in the treated region and restrict its diffusion into neighboring areas. Our results showed that KGN implantation not only resulted in the disorganization of collagen fibers, and induced hyper-cellularity and vascularity at the implanted site, but also produced chondrocyte differentiation, and increased proteoglycan accumulation and Col. II expression, which are typical features of over-use tendinopathy created by long term treadmill running in rat supraspinatus tendons [26,27]. Therefore, this KGN-bead implantation creates an animal model of tendinopathy similar to that induced by treadmill running. Such KGN-induced animal tendinopathy model also captures certain features of degenerative tendinopathy frequently observed in clinics, including the disorganization of collagen fibers and formation of cartilage-like tissues in tendon lesions [28–31].

Recent studies have implicated the primary role of TSCs in the development of tendinopathy [9,23,32]. TSCs are a newly discovered cell type in the tendons of mice, rats, rabbits and humans [33–35], and growing evidence suggests their dual role in tendon homeostasis and pathology. TSCs also play a critical role in maintaining tenocyte numbers in normal tendons [33]. However, their role in the etiology of tendinopathy is only now emerging. In previous studies, we established that over-use tendinopathy induced *in vivo* by treadmill running or *in vitro* by mechanical stretching was largely caused by the aberrant differentiation of TSCs into non-tenocytes such as chondrocytes, osteocytes and adipocytes [9]. To investigate whether TSCs are also involved in the KGN induced tendinopathy *in vivo*, we performed an *in vitro* study to evaluate the effects of KGN on TSC differentiation. The results indicated that KGN can induce chondrogenic differentiation by increasing the accumulation of glycosaminoglycans in TSCs cultured with various KGN concentrations (**Fig 8**). This suggests that the observed KGN-induced chondrogenic differentiation in rat tendons *in vivo* (**Fig 2**) is likely due to the effects of KGN on TSCs. It should be noted that during implantation of KGN-beads, a small wound (or a small puncture) was created in the rat tendons in order to secure the KGN-bead in place. Because of such injury, other types of stem cells, such as circulating bone marrow mesenchymal stem cells (BMSCs) could have migrated to the wound site and as a result, participated in the KGN-induced chondrogenic differentiation, in addition to TSCs. Our previous showed that KGN can also increase the chondrocyte differentiation of BMSCs [23]. Therefore, it is likely that circulating BMSCs could have contributed to the chondrogenic differentiation observed *in vivo* in rat Achilles tendons. However, because the wound was minimal and tendons are not highly vascularized tissues, the contribution of these BMSCs to such chondrogenic differentiation seen in this animal model of tendinopathy may be minimal when compared to local TSCs. Future study is warranted to investigate the specific contributions of TSCs and other stem cells on chondrogenesis.

Our previous study also showed that lower concentrations of KGN (1–5 μM) induced similar effects in rat TSCs [23]. These findings indicate that TSC based mechanisms in part, induce the "features" of over-use tendinopathy in our KGN induced tendinopathy model. Therefore, the animal model of tendinopathy created by KGN implantation is "mechanistic," that is, it is based on TSC’s chondrogenic differentiation, similar to conditions where TSCs are subjected to large mechanical loading, which induces their differentiation into non-tenocyte lineages of cells [32].

Recently, Johnson et al [20] showed that the action mechanism of KGN involved upregulating the transcription factor, Runx-1. Injection of KGN in osteoarthritic mice knees resulted in KGN binding to filamin A, an actin-binding protein that crosslinks actin filaments [36]. This binding disrupts the association between filamin A and the transcription factor, CBFβ, thus translocating CBFβ from the cytoplasm to the nucleus that subsequently increases Runx-1, which is normally up-regulated only in chondrocytes [20]. Thus, the increase of Runx-1 caused by KGN is likely responsible for the appearance of more chondrocytes in our KGN-induced tendinopathy model because Runx-1 has been shown to play a critical role in chondrogenesis and chondrocyte proliferation [37]. Future studies will determine the effects of KGN on the signaling pathways involved in rat tendons.

In this study, we used alginate gel as a carrier to deliver KGN directly into the rat Achilles tendon. It has been reported that chondrocyte-calcium alginate gel constructs could generate cartilage-like tissues after implantation into the subcutaneous pockets of nude mice [38]. Therefore, calcium alginate gels would be suitable delivery systems for the encapsulation and transplantation of KGN.

Alginate forms a viscous solution when dissolved in saline and becomes a gel on contact with divalent cations. In addition, alginate beads can preserve the viability and phenotype of chondrocytes [39]. This alginate-KGN bead could allow the controlled-release of KGN into the expected location to provide an effective concentration of KGN for a prolonged time and avoid drug diffusion. Indeed, our *in vitro* KGN release study showed that KGN is released continuously from beads (**Fig 7**). These *in vitro* test results suggest the likelihood of KGN release after KGN-bead implantation *in vivo* at the implantation site causing TSCs and possibly other types of stem cells (e.g. BMSCs) to differentiate into chondrocytes thus forming cartilage-like tissues in the tendon.

### Summary

We showed in this study that by using KGN-bead implantation, it is feasible to create an animal model of tendinopathy that exhibits localized tendinopathic lesions that are similar to those created by long term rat treadmill running [26] and also capture certain degenerative features of clinical tendinopathy [31]. Compared to existing tendinopathy models, this new animal model of tendinopathy has the following advantages: 1) it is cost-effective and efficient (reduces animals and time); 2) it is highly reproducible and 3) it can create *localized* tendinopathic lesions at any region of the tendon such as insertional tendinopathy [40,41], which is common in clinics and difficult to reproduce by other means such as treadmill running. Such a localized tendinopathy model can be useful to study the effects of various treatments on tendinopathy. For example, it can be used to evaluate the localized effects of various drugs such as NSAIDs in the treatment of various stages of tendinopathy. Additionally, it can be used to assess the efficacy of other treatment options, e.g. PPR, which is widely used to treat tendinopathy by injection in clinics [42–44].

## References

[1] Xu Y, Murrell GAC (2008) The Basic Science of Tendinopathy. Clin Orthop Relat Res 466: 1528–1538. 10.1007/s11999-008-0286-4 18478310 PMC2505234

[2] Wang JH, Iosifidis MI, Fu FH (2006) Biomechanical basis for tendinopathy. Clin Orthop Relat Res 443: 320–332. 16462458 10.1097/01.blo.0000195927.81845.46

[3] Aström M, Rausing A (1995) Chronic Achilles tendinopathy. A survey of surgical and histopathologic findings. Clin Orthop Relat Res 316: 151–164. 7634699

[4] Corps AN, Robinson AHN, Movin T, Costa ML, Hazleman BL, Riley GP (2006) Increased expression of aggrecan and biglycan mRNA in Achilles tendinopathy. Rheumatology 45: 291–294. 16219640 10.1093/rheumatology/kei152

[5] Samiric T, Parkinson J, Ilic MZ, Cook JL, Feller JA, Handley CJ (2009) Changes in the composition of the extracellular matrix in patellar tendinopathy. Matrix Biology 28: 230–236. 10.1016/j.matbio.2009.04.001 19371780

[6] Parkinson J, Samiric T, Ilic MZ, Cook J, Feller JA, Handley CJ (2010) Change in proteoglycan metabolism is a characteristic of human patellar tendinopathy. Arthritis & Rheumatism 62: 3028–3035. 10.1002/art.27587 20533294

[7] Jiang D, Wang JH (2013) Tendinopathy and its treatment with platelet-rich plasma (PRP). Histol Histopathol 28: 1537–1546. 23857144 10.14670/HH-28.1537

[8] Fung DT, Wang VM, Andarawis-Puri N, Basta-Pljakic J, Li Y, Laudier DM, et al (2010) Early response to tendon fatigue damage accumulation in a novel in vivo model. J Biomech 43: 274–279. 10.1016/j.jbiomech.2009.08.039 19939387 PMC3366582

[9] Zhang J, Wang JH (2013) The Effects of Mechanical Loading on Tendons—An In Vivo and In Vitro Model Study. PLoS ONE 8: e71740 10.1371/journal.pone.0071740 23977130 PMC3747237

[10] Glazebrook MA, Wright JR Jr., Langman M, Stanish WD, Lee JM (2008) Histological analysis of achilles tendons in an overuse rat model. J Orthop Res 26: 840–846. 10.1002/jor.20546 18183626

[11] Ng GY-F, Chung PY-M, Wang JS, Cheung RT-H (2011) Enforced Bipedal Downhill Running Induces Achilles Tendinosis in Rats. Connective Tissue Research 52: 466–471. 10.3109/03008207.2011.562334 21591929

[12] Silva RD, Glazebrook MA, Campos VC, Vasconcelos AC (2011) Achilles tendinosis: a morphometrical study in a rat model. Int J Clin Exp Pathol 4: 683–691. 22076169 PMC3209609

[13] Abraham T, Fong G, Scott A (2011) Second harmonic generation analysis of early Achilles tendinosis in response to in vivo mechanical loading. BMC Musculoskelet Disord 12: 1471–2474. 10.1186/1471-2474-12-26 PMC3045393 21269488

[14] Stone D, Green C, Rao U, Aizawa H, Yamaji T, Niyibizi C, et al (1999) Cytokine-induced tendinitis: A preliminary study in rabbits. Journal of Orthopaedic Research 17: 168–177. 10221832 10.1002/jor.1100170204

[15] Sullo A, Maffulli N, Capasso G, Testa V (2001) The effects of prolonged peritendinous administration of PGE1 to the rat Achilles tendon: a possible animal model of chronic Achilles tendinopathy. J Orthop Sci 6: 349–357. 11479765 10.1007/s007760100031

[16] Khan MH, Li Z, Wang JH (2005) Repeated exposure of tendon to prostaglandin-e2 leads to localized tendon degeneration. Clin J Sport Med 15: 27–33. 15654188 10.1097/00042752-200501000-00006

[17] Marsolais D, Cǒté CH, Frenette J (2001) Neutrophils and macrophages accumulate sequentially following Achilles tendon injury. Journal of Orthopaedic Research 19: 1203–1209. 11781025 10.1016/S0736-0266(01)00031-6

[18] Hsu RW-W, Hsu W-H, Tai C-L, Lee K-F (2004) Effect of shock-wave therapy on patellar tendinopathy in a rabbit model. Journal of Orthopaedic Research 22: 221–227. 14656684 10.1016/S0736-0266(03)00138-4

[19] Bell R, Li J, Gorski DJ, Bartels AK, Shewman EF, Wysocki RW, et al (2013) Controlled treadmill exercise eliminates chondroid deposits and restores tensile properties in a new murine tendinopathy model. J Biomech 46: 498–505. 10.1016/j.jbiomech.2012.10.020 23159096

[20] Johnson K, Zhu S, Tremblay MS, Payette JN, Wang J, Bouchez LC, et al (2012) A stem cell-based approach to cartilage repair. Science 336: 717–721. 10.1126/science.1215157 22491093

[21] Fu S-C, Chan K-M, Rolf CG (2007) Increased Deposition of Sulfated Glycosaminoglycans in Human Patellar Tendinopathy. Clinical Journal of Sport Medicine 17: 129–134 110.1097/JSM.1090b1013e318037998f. 17414481 10.1097/JSM.0b013e318037998f

[22] Da Violante G, Zerrouk N, Richard I, Provot G, Chaumeil JC, Arnaud P (2002) Evaluation of the cytotoxicity effect of dimethyl sulfoxide (DMSO) on Caco2/TC7 colon tumor cell cultures. Biol Pharm Bull 25: 1600–1603. 12499647 10.1248/bpb.25.1600

[23] Zhang J, Wang JH (2014) Kartogenin induces cartilage-like tissue formation in tendon-bone junction. Bone Res 2. 10.1038/boneres.2014.8 PMC4237211 25419468

[24] Livak KJ, Schmittgen TD (2001) Analysis of Relative Gene Expression Data Using Real-Time Quantitative PCR and the 2−ΔΔCT Method. Methods 25: 402–408. 11846609 10.1006/meth.2001.1262

[25] Stone D, Green C, Rao U, Aizawa H, Yamaji T, Niyibizi C, et al (1999) Cytokine-induced tendinitis: a preliminary study in rabbits. J Orthop Res 17: 168–177. 10221832 10.1002/jor.1100170204

[26] Scott A, Cook JL, Hart DA, Walker DC, Duronio V, Khan KM (2007) Tenocyte responses to mechanical loading in vivo: a role for local insulin-like growth factor 1 signaling in early tendinosis in rats. Arthritis Rheum 56: 871–881. 17328060 10.1002/art.22426

[27] Archambault JM, Jelinsky SA, Lake SP, Hill AA, Glaser DL, Soslowsky LJ (2007) Rat supraspinatus tendon expresses cartilage markers with overuse. J Orthop Res 25: 617–624. 17318892 10.1002/jor.20347

[28] Khan KM, Cook JL, Bonar F, Harcourt P, Astrom M (1999) Histopathology of common tendinopathies. Update and implications for clinical management. Sports Med 27: 393–408. 10418074 10.2165/00007256-199927060-00004

[29] Cetti R, Junge J, Vyberg M (2003) Spontaneous rupture of the Achilles tendon is preceded by widespread and bilateral tendon damage and ipsilateral inflammation: a clinical and histopathologic study of 60 patients. Acta Orthop Scand 74: 78–84. 12635798 10.1080/00016470310013707

[30] Buck FM, Grehn H, Hilbe M, Pfirrmann CW, Manzanell S, Hodler J (2009) Degeneration of the long biceps tendon: comparison of MRI with gross anatomy and histology. AJR Am J Roentgenol 193: 1367–1375. 10.2214/AJR.09.2738 19843755

[31] Kannus P, Jozsa L (1991) Histopathological changes preceding spontaneous rupture of a tendon. A controlled study of 891 patients. J Bone Joint Surg Am 73: 1507–1525. 1748700

[32] Zhang J, Wang JH (2010) Mechanobiological response of tendon stem cells: implications of tendon homeostasis and pathogenesis of tendinopathy. J Orthop Res 28: 639–643. 10.1002/jor.21046 19918904

[33] Bi Y, Ehirchiou D, Kilts TM, Inkson CA, Embree MC, Sonoyama W, et al (2007) Identification of tendon stem/progenitor cells and the role of the extracellular matrix in their niche. Nat Med 13: 1219–1227. 17828274 10.1038/nm1630

[34] Zhang J, Wang JH (2010) Characterization of differential properties of rabbit tendon stem cells and tenocytes. BMC Musculoskelet Disord 11: 10 10.1186/1471-2474-11-10 20082706 PMC2822826

[35] Rui YF, Lui PP, Li G, Fu SC, Lee YW, Chan KM (2010) Isolation and characterization of multipotent rat tendon-derived stem cells. Tissue Eng Part A 16: 1549–1558. 10.1089/ten.TEA.2009.0529 20001227

[36] Gorlin JB, Yamin R, Egan S, Stewart M, Stossel TP, Kwiatkowski DJ, et al (1990) Human endothelial actin-binding protein (ABP-280, nonmuscle filamin): a molecular leaf spring. J Cell Biol 111: 1089–1105. 2391361 10.1083/jcb.111.3.1089 PMC2116286

[37] Wang Y, Belflower RM, Dong Y-F, Schwarz EM, O'Keefe RJ, Drissi H (2005) Runx1/AML1/Cbfa2 Mediates Onset of Mesenchymal Cell Differentiation Toward Chondrogenesis. Journal of Bone and Mineral Research 20: 1624–1636. 16059634 10.1359/JBMR.050516

[38] Paige KT, Cima LG, Yaremchuk MJ, Schloo BL, Vacanti JP, Vacanti CA (1996) De novo cartilage generation using calcium alginate-chondrocyte constructs. Plast Reconstr Surg 97: 168–178. 8532775 10.1097/00006534-199601000-00027

[39] Riley G (2004) The pathogenesis of tendinopathy. A molecular perspective. Rheumatology (Oxford) 43: 131–142. 12867575 10.1093/rheumatology/keg448

[40] Kearney R, Costa ML (2010) Insertional Achilles Tendinopathy Management: A Systematic Review. Foot & Ankle International 31: 689–694. 20727317 10.3113/FAI.2010.0689

[41] Krishna Sayana M, Maffulli N (2005) Insertional Achilles Tendinopathy. Foot and Ankle Clinics 10: 309–320. 15922921 10.1016/j.fcl.2005.01.010

[42] Mishra A, Pavelko T (2006) Treatment of chronic elbow tendinosis with buffered platelet-rich plasma. Am J Sports Med 34: 1774–1778. 16735582 10.1177/0363546506288850

[43] Yuan T, Zhang CQ, Wang JH (2013) Augmenting tendon and ligament repair with platelet-rich plasma (PRP). Muscles Ligaments Tendons J 3: 139–149. 24367773 PMC3838322

[44] Wang JH (2014) Can PRP effectively treat injured tendons?. Muscles Ligaments Tendons J 4: 35–37. 24932445 PMC4049648

